# Men’s experiences of decision-making in life-prolonging treatments of metastatic castration-resistant prostate cancer – wishing for a process adapted to personal preferences: a prospective interview study

**DOI:** 10.1186/s12911-025-02985-x

**Published:** 2025-03-31

**Authors:** Sandra Doveson, Agneta Wennman-Larsen, Per Fransson, Lena Axelsson

**Affiliations:** 1 Department of Nursing Science, Sophiahemmet University, Lindstedtsvägen 8, PO Box 5605, Stockholm, SE-114 86 Sweden; 2 Department of Health Care Sciences, Marie Cederschiöld University, PO Box 11189, Stockholm, SE- 100 61 Sweden; 3 Division of Insurance Medicine, Department of Clinical Neuroscience, Karolinska Institutet, Berzelius väg 3, Stockholm, SE-171 77 Sweden; 4 Department of Nursing, Umeå University, Umeå, SE- 901 87 Sweden

**Keywords:** Castration-resistant, Communication, Decision-making, Nurse-patient relations, Oncology nursing, Prostate neoplasm, Therapeutics

## Abstract

**Background:**

In the fast-expanding field of life-prolonging-treatment of metastatic, castration-resistant prostate cancer, treatment decision-making is very complex - both for patients and healthcare professionals since there is no “one size that fits all” in choosing treatment in this phase. Little research has been conducted about men’s experiences of treatment decision-making in this advanced, incurable, phase. Hence, this study aimed to describe men’s experiences of decision-making in life-prolonging treatments of metastatic castration-resistant prostate cancer.

**Methods:**

Seventeen men were recruited from four oncology clinics in Sweden and interviewed at baseline. Qualitative interviews (*n* = 31) were conducted over two years, the timepoints for subsequent interviews (10 men were interviewed twice or more) adhered to when each man switched or terminated life-prolonging treatment. Data was analysed with qualitative content analysis.

**Results:**

Initially, the men were adamant about proceeding with treatment. As their illness continued to progress, they gradually turned their focus more towards their well-being. They wished for continuity regarding treating physicians and constantly being assigned new physicians compromised the quality of care and complicated decision-making. In their decision-making, the men adapted their own approach to the approach taken by their physician, even if it was not an approach they had originally preferred. They wished for their role preferences to be respected. Most men had made treatment decisions collaboratively with their physician, but some described having taken on a more, or less, driving role in decision-making than they really wished for. Navigating healthcare was perceived as difficult and for some it thus felt necessary to pursue and coordinate their own care by e.g. using personal connections or contacting clinics ahead of referral. A part of treatment decision-making was forming a basis for a decision, in which the need for personalized information (quality, quantity and timing) came forth as important.

**Conclusions:**

When diagnosed with metastatic castration-resistant prostate cancer, men’s preferences for their decision-making role, and perspectives on the treatment outcome need to be continuously addressed throughout their disease course. Improved continuity of care and a more personalised care approach should meet these patients’ wishes and needs in this phase.

**Trial registration:**

Clinical trial number: Not applicable.

**Supplementary Information:**

The online version contains supplementary material available at 10.1186/s12911-025-02985-x.

## Background

Prostate cancer (PC) is one of the most common cancers and leading causes of cancer-related death globally [1], as well as the most common cancer among men in Sweden [2]. Most of the men are diagnosed with localised disease, for which the treatment options include curatively intended prostatectomy or radiation therapy. For low-risk PC, active surveillance is recommended with systematic follow ups for eventual later curative treatment [3]. Men’s decision-making in localised PC has been thoroughly studied and preferences for their own role in treatment decision-making have been found to be diverse. Shared decision-making (where the patient and physician make the decision together) is preferred by many [4–7], but there are also findings showing that some men prefer to either make the treatment decision more independently [4, 5, 8] or that the physician makes the decision for them [4, 5, 9]. The treating physician [10, 11] and partners/families [12] are important influencers on which treatment they eventually choose.

A small proportion of men with PC are, however, diagnosed with metastatic disease and about 15–30% of men with localised disease develop metastases [13]. Metastatic PC (mPC) is not curable, but treatment may inhibit disease progression [14]. Similarly to localised PC, the physician has been found to be a key factor in treatment decision-making among men with mPC. The physician’s treatment recommendation has been reported as the single most important factor for men when they are considering a treatment, closely followed by a wish to feel well enough to be able to spend time with their loved ones [15]. Even though research on the structure of treatment decision-making in men with mPC is scarce, it has been shown that, like men in the localised phase [6, 7], most men with mPC report having had a shared treatment decision-making experience [16]. The agreement between the men and their physicians regarding how the treatment decision had been made (shared, physician-driven, or patient-driven) was, however, low. An agreement about how the decision had been made was seen in less than 50% of the cases, indicating the need to explore patients’ preferences for the structure of treatment decision-making to enable for participation in a preferred way [16].

Over time, men with mPC may become resistant to the hormone treatment and the PC reaches its most advanced stage – metastatic castration-resistant prostate cancer (mCRPC), for which the prognosis is poor with limited survival [14]. mCRPC also comes with deteriorating health-related quality of life (QoL) [17] and severe symptoms [18]. Since 2004, however, the life-prolonging treatment options have increased and today there are several options for men with mCRPC [14]. Patients may receive one or several lines of treatment, including options such as chemotherapy, hormone treatments and radionuclide treatment [14], for which possible side effects include neutropenia, fatigue, alopecia, nausea/vomiting, sensory neuropathy, diarrhea [19], fluid retention, edema, hypokalemia, hypertension, cardiac events [20], hot flashes, musculoskeletal pain, and headache [21]. As the treatments and sequencing options for mCRPC increase, treatment decision-making becomes altogether more difficult both for the man and for the treating physician. Moreover, since there is not one single treatment sequence that serves as the gold standard for mCRPC, considerations must be taken to e.g. the patient’s performance status, comorbidities, symptoms, outcomes of previous mCRPC-directed treatments and patient treatment preference, both when starting and when switching life-prolonging treatment [14]. Due to this complex situation, it is recommended to have a specialist nurse in the prostate cancer care team to support in treatment decision-making, and to participate in follow-ups [22]. Patients who undergo nurse-led follow-ups in prostate cancer care have also been shown to be equally satisfied with their care as patients who undergo urologist-led follow-ups [23, 24].

Among men with mCRPC specifically, research shows that they weigh treatment benefits against risks when they are facing life-prolonging treatments but also express acceptance of discomfort in hope of prolonging life [25]. Similar results have been shown among patients with other incurable cancers [26]. Further, a discrepancy has been found between patients’ and healthcare professionals’ priorities in treatment decision-making in a study across all stages of various cancers. Patients commonly prioritised survival over QoL, whereas their QoL often was found to be regarded as more important by healthcare professionals [27]. Another aspect of the complexity in treatment decision-making in the late phases of cancer is the potential treatment side effects that could impact QoL and everyday lives. In men with mCRPC specifically, research shows that QoL could be seen as more important than maximizing life expectancy if it comes at the cost of debilitating treatment-related side effects [28]. Even though the research field of treatment decision-making in cancer is growing, the findings on priorities of patients and physicians regarding QoL, treatment side effects and survival does not necessarily apply to all patients and physicians and discussions on individual levels are always needed.

In summary, mCRPC comes with increasing challenges related to treatment decision-making with dramatically increased treatment possibilities over the past decade. Further, in this incurable disease phase, quality of life has to be balanced against potential survival benefits of treatment. Given what has been shown earlier in the disease course, it is obvious that men’s experiences of treatment decision-making and their preferences for their treatment decision-making role cannot be assumed but needs to be further explored also in men with mCRPC.

## Methods

### Aim

This study aimed to describe men’s experiences of treatment decision-making in life-prolonging treatments of mCRPC.

### Design

This study has a prospective, qualitative descriptive design with an inductive approach using serial interviews [29].

### Procedure, setting and participants

The present study is part of a prospective, multicentre research project focusing on men’s experiences when undergoing life-prolonging treatment(s) of mCRPC. The inclusion criteria for the research project were:

- men who had been diagnosed with mCRPC and were planned to undergo life-prolonging treatment, regardless of type of life-prolonging treatment.
- men who were able to understand and express themselves in Swedish.

The men were recruited consecutively from four oncology clinics in Sweden. They were given information about the study and were asked to participate when they had been diagnosed with mCRPC and scheduled to start their first life-prolonging treatment. All included participants provided written informed consent. In total, 154 men with mCRPC were included in the overall research project, from which the present study derived a sub-sample for qualitative interviewing.

Purposive sampling was applied to select the sub-sample for this interview study, in order to achieve credibility and include participants with various experiences and rich information [30]. A variation was sought regarding place of residence, age, relationship status, educational level, place of birth and type of first life-prolonging treatment. A tentative sample size for the present study was set at 15–20 participants, since we wished to follow each participant’s unique treatment trajectory. A research nurse/coordinator approached 19 participants in the study and asked them for permission for a researcher to contact them regarding the interview study. All nineteen agreed and were telephoned by a researcher, received information about the interview study, and were asked if they were interested in participating. Seventeen of the men agreed while two declined due to ill health and not feeling well enough.

The participating men resided in rural and urban areas in various parts of Sweden. Their ages ranged from 60 to 82 years (mean: 73 years). Fourteen men had a spouse, three were not in a relationship. The participants’ education ranged from nine-year compulsory school to university-level studies, the majority (82%) had completed high school-level studies or higher. All were born in Sweden. Fifteen men scored $\:\le\:1$ on the ECOG Performance Status [31] (Table 1). Chemotherapy (Docetaxel) and hormone treatments (Abiraterone; Enzalutamide) were represented among the men’s first life-prolonging treatments and chemotherapy (Cabazitaxel), hormone treatments (Abiraterone; Enzalutamide) and Radium-223 were represented among the consecutive treatment lines (Table 2).

**Table 1:** Participant characteristics at baseline

Participant characteristics at baseline	Total No. (*N* = 17)
Age in years	
Mean/Median	73/75
(range)	(60-82)
ECOG performance status	*n*
0	8
1	7
2	1
3	1
Relationship status	
In a relationship	14
Not in a relationship	3
Educational level	
9-year compulsory school	3
High school	6
University	8
Place of birth	
Sweden	17
Other Nordic country	0
Other European country	0
Country outside of Europe	0
Occupation	
Working part time	1
On sickness absence from work	1
Retired	15

**Table 2:** Distribution of life-prolonging treatments among the men over time

	1st treatment	2nd treatment	3rd treatment	4th treatment	5th treatment	Total
Docetaxel	12	-	-	-	-	12
Abiraterone	1	1	-	-	-	2
Enzalutamide	4	5	-	-	-	9
Radium-223	-	1	-	-	-	1
Cabazitaxel	-	-	3	1	-	4
N/A; N/K	-	-	-	2	1	3
Total						31

### Data generation

Seventeen men were interviewed as they were either about to start, currently undergoing or had completed their first life-prolonging treatment. The men were thereafter offered participation in interviews in conjunction with either the start of additional, or the definite termination of, life-prolonging treatments. A total of 31 qualitative interviews [29] were performed between 2016 and 2019. Seventeen men were interviewed at baseline. Seven out of those did not start any additional treatment(s) during their time in the study, and were, hence, only interviewed once. Two men declined to participate in further interviews following the first one, but did not provide a reason for their decision. One man did start a second treatment but was lost to follow up and was therefore only interviewed once. Seven men proceeded with new treatments and completed a second interview, three men completed a third and fourth interview and one man completed five interviews. The declining number of interviews over time is due to the participants not switching, nor terminating, life-prolonging treatment (Table 3).

**Table 3:** Interviews in conjunction with change of life-prolonging treatments over time

Participant	1st treatment	2nd treatment	3rd treatment	4th treatment	5th treatment	Total
**1**	X	-	-	-	-	1
**2**	X	0	-	-	-	1
**3**	X	0	0	-	-	1
**4**	X	X	-	-	-	2
**5**	X	-	-	-	-	1
**6**	X	0	-	-	-	1
**7**	X	-	-	-	-	1
**8**	X	-	-	-	-	1
**9**	X	-	-	-	-	1
**10**	X	X	X	X	X	5
**11**	X	X	-	-	-	2
**12**	X	-	-	-	-	1
**13**	X	X	X	X	-	4
**14**	X	X	-	-	-	2
**15**	X	-	-	-	-	1
**16**	X	X	-	-	-	2
**17**	X	X	X	X	-	4
	17	7	3	3	1	31

X Interviewed; 0 Not interviewed; - No new life-prolonging treatment;

The interviews were performed face-to-face (*n* = 28) or via telephone (*n* = 3) depending on the men’s wishes. All were conducted by the first author, except for two interviews conducted by the second author and a research nurse respectively. To encourage participants to narrate freely, the baseline interviews commenced with the question: “Would you like to tell me about your situation with prostate cancer?” and the follow-up interviews opened with “Would you like to tell me how you have been since we last saw each other?”, using a thematic interview guide developed for this study (Supplementary 1). Depending on what topics came up, questions were thereafter formed to follow up, or probe them further and to encourage the men to narrate various dimensions of their experiences of treatment decision-making. Brief notes were taken to help pick up on or return to topics during interviews. The total time of the audio recorded interviews was 32 h and 19 min (range 22–160 min, median = 65 min).

Sixteen of the baseline interviews also constitute data for another study within the research project [25], that focused on men’s expectations and perspectives when they are faced with a life-prolonging treatment. During these 16 interviews, data was generated for both the present study and the previous one [25] using an interview guide that was developed to cover the aims of both studies. Following the 16 baseline interviews, data generation then continued for the present study and another 15 interviews were conducted focusing only on treatment decision-making.

### Data analysis

Qualitative inductive content analysis was used as a method to systematically analyse data responding to the study aim, allowing interpretation of different depths [32, 33]. Firstly, all interviews were transcribed verbatim. They were then read and listened to several times by the first author to develop a sense of the data and content as a whole. The first part of the analysis was performed by the first author in collaboration with the last author, who also read all interviews. The study aim guided the identification of meaning units, i.e. segments in the text relating to experiences of decision-making. All meaning units were thereafter condensed, meaning they were shortened while still preserving the core. Following condensation, the meaning units were coded and the codes were used as tools to search for similarities, differences, and patterns among the meaning units. Codes relating to each other were clustered into tentative descriptive subthemes and -themes. A descriptive theme conveys nuances within the data and provide answers to questions such as: “what is going on here?” and “What are the participants trying to tell me?” [33]. Meaning units from each participant were also compared over time within all subthemes and themes. The subthemes and themes were discussed among all authors on several occasions and then finalised. Throughout the analysis process, the authors moved back and forth between the different steps in the analysis to maintain a close connection between the codes and their context in the interview.

### Ethical considerations

The study was performed according to the ethical principles stated in the Declaration of Helsinki [34] and approved by the Regional Ethical Review Board (now the Swedish Ethical Review Authority) in Stockholm, Sweden (Dnr 2014/341 − 31/2, Dnr 2016/851 − 32 and Dnr 2016/2230-32).

## Results

The results consist of four main themes with eight subthemes (Table 4).

**Table 4:** Overview of themes with related subthemes

Themes	Subthemes
Making treatment decisions within an incurable illness frame	Making treatment decisions experiencing a silent illness
	Being confronted with the gravity of one’s illness
Changing perspectives on the treatment outcome	-
Adapting to different decision-making roles and relations	Modifying one’s own decision-making role
	Seeking trust and continuity in the patient-physician relationship
	Navigating an unpredictable healthcare organisation
Forming a basis for treatment decisions	Being guided by beliefs about cancer and cancer treatments
	Wanting and looking for personalised information
	Waiting for a treatment evaluation

### Making treatment decisions within an incurable illness frame

#### Making treatment decisions experiencing a silent illness

Prostate cancer came forth as a silent illness as some men explained that they had experienced no symptoms before the diagnosis or even throughout the disease trajectory, as described by one man:

*I can’t really say that … the cancer (…) affects my daily life. I don’t feel it. I don’t know if I have … well, I DO know that I have it but I … I don’t know, I don’t feel it.* – Man G.

They found it strange and almost unreal having to make a treatment decision without having ever felt an illness that had instead been detected through a prostate-specific antigen (PSA) test. One man described a conversation he had with his physician:

*It’s very odd with you, he said, because … we’re dealing with … a lab value, since you don’t feel your cancer at all, so we’re trying to fix something that is a lab value.* – Man A.

At times, the illness continued to act silently and hence, they made treatment decisions without feeling ill.

#### Being confronted with the gravity of one’s illness

When receiving the mCRPC diagnosis and being confronted with the gravity of their illness, the men described how they felt like their life was threatened when faced with treatment decisions:

*No I thought … well, I’m in my mid-seventies so … all my friends have … (…) they were amazing friends. Now they’re … gone, huh. There’s one … there’s two left out of eight. And it was cancer and all sorts of things they passed away from, they didn’t … they didn’t make it to seventy. So … then maybe now it’s … it’s my turn. So I was prepared for that, for it to be over.* – Man I.

They talked about their hopes in terms of the treatment “putting the brakes on cancer” or “slowing it down”. In light of their life being threatened, the decision to choose the most effective treatment was first described as easy to make, as possible treatment side effects were viewed as less significant compared to possibly living longer:

*I had medication and … or chemotherapy to choose in between but … it was nothing … to choose between since chemotherapy was the most effective (…) I thought that I’ll have to take it and feel lousy for a while just so … I can get this poison in me and it slows it [the cancer] down.* – Man E.

When learning that the cancer had continued to grow despite ongoing treatment, they described knowing that their prognosis and treatment possibilities had changed for the worse. This was shocking for some since they had had high hopes for the treatment. A failed treatment also involved yet another decision – whether to proceed with new treatment or not to treat at all.

### Changing perspectives on the treatment outcome

The men’s treatment decision-making was related to their changing perspectives on the treatment outcome. The men described a balancing act between wanting to prolong life and the treatment intrusion. During their first interview, most proclaimed that they were willing to endure severe side effects as a necessary price to “buy more time”. In hindsight, in the follow-up interviews, most still described feeling content with the decision to treat. However, some men felt uncertain as to whether going through with the treatment had been worth it:

*What I’ve been experiencing from the start is, (…) what the difference is for me if I don’t get it [treatment] and what effect it will have? Because in hindsight now, I sometimes wonder if I really would have gone through all this [treatments]. But I don’t really know the option.”* – Man J.

The men’s previous treatment experiences also conveyed into new decisions, e.g., experiences of severe side effects from chemotherapy could make them more hesitant to consider this again. They focused more on their well-being and the content of their life and reflected on whether going ahead with a new treatment would allow them to live as they wanted:

*And for me, (…) I’ve been thinking a lot about how I want quality during that time, I don’t wanna live just to survive. That’s easy to say and I don’t know if I’m making those decisions but that’s the way I’ve been thinking. To choose just to live another month and feel like crap … because I’m thinking … I’d rather feel good until ‘bam’ [I die].* – Man J.

At this point, the men could also ask their physician for a treatment break:

*When they saw the [PSA] levels, that they’d gone up again, (…) then we talked about this, (…). ‘ I think’, I said, ’ that if it’s possible … then we’ll wait for it until August, huh’, I believe I said, ‘so that I can have some peace and quiet over the summer and not be running up here [at the clinic]’.* – Man H.

Otherwise, treatment breaks were usually initiated by the physician when the outcome was not as hoped, or due to severe side effects. However, there were also men who expressed that they had still wanted to proceed with the treatment had the decision been theirs, as they worried that the cancer would grow out of control. Others instead expressed how they secretly felt relieved that their physician decided to cease the specific treatment as they would be given a window to recuperate.

### Adapting to different decision-making roles and relations

#### Modifying one’s own decision-making role

The men modified their own decision-making roles, meaning their roles could vary. They described this as primarily influenced by the unique meetings with different physicians. Some described being presented with the one treatment their physician regarded as best for them in their current illness situation and felt confident that this was the best option. Others instead would have wanted a discussion with a comparison of different options and were disappointed when this did not occur, or their request for it was dismissed:

*There were several times when I was … eehrm … disappointed, that we didn’t have more of a conversation. One was summoned for a conversation but then the conversation didn’t happen. (…) They wanted to know how I was doing and so on, but … they didn’t … they didn’t have any detailed information about my condition and … what it [treatment] looked like going forward and things like that.* – Man I.

With certain physicians, some men had a collaborative decision-making experience where they discussed feasible treatment options, and then decided on a treatment plan together, which they appreciated. Others had suggested a treatment to the physician after investigating options themselves. Whereas a group of men described getting approval, others had experienced being denied their suggestions. However, they agreed to go with the physician’s suggestion, hoping that it really was the best option. Some described having found decision-making advice or support in close persons such as family members or friends. However, most men described that they nonetheless made the subsequent treatment decision with the physician or independently.

Among the men asked by their physician about treatment preferences, some found it odd that their opinion was assigned such weight. To them, the physician was an experienced oncologist whereas they themselves were laymen. When asked about their preference, some sought guidance by asking their male physician what treatment he would have chosen for himself:

*I said. ‘If you’d been in my shoes, what would you have chosen?’ He [the physician] thought for a second and then he said ‘Then I’d take [medication]’. (…) ‘Great’, I said, ‘then let’s go with that.’* – Man A.

The men reflected upon what participation in treatment decision-making constituted. To them, being an active participant did not necessarily mean wanting to be the deciding party, as partaking in decision-making could mean being well-informed but still wanting the physician to take the final decision:

*They keep talking about how patients should participate and almost decide about their … their care. And I don’t really get that. I don’t think I’m competent to … decide anything about my care.*

I: What are your thoughts about that?

*I think it’s perfectly ok that I get the information and answers to the questions I have, but for me to sit and decide whether I should start chemotherapy or not, that’s … I’ll leave that for the experts to decide. They have the knowledge. I’m not an oncologist.* – Man C.

Regardless of specific decision-making role preference, the most important concern was that the man’s preference was respected.

#### Seeking trust and continuity in the patient-physician relationship

Trusting one’s physician was key to how the men positioned themselves when treatment decisions were made. The men who had experienced continuity underlined that this enabled trust and security in the relationship, which facilitated a dialogue about treatment options and goals. Several men, however, described constantly being assigned different physicians which complicated decision-making since the physician did not know them or their priorities, as expressed by one man:

*I got new doctors every time, despite telling them that I wanted the same doctor. I didn’t want one that’s gonna read about me just before [the appointment]. Besides, you didn’t know if they HAD read about you. But that … didn’t happen. (…) At a couple of occasions it was even like this … ‘drive-in-service’, that I call it, which means you’re seated in a row … outside the doctors. And then the nurses came and put your chart in the doctor’s box, they [the physician] reached out and got the chart … and two minutes later you were … you were called in. There’s no doctor that can familiarize themselves with these cases like that, it was pathetic.* – Man G.

The lack of continuity also constituted a barrier to ask questions or talk about their lives and wishes. Sensing a genuine interest and commitment from their physician was crucial for the men in this life-threatening situation:

*When you think it [the PSA value] goes like this [rises rapidly], then it doesn’t feel good, huh. (…) And then … my feeling that … this isn’t his [the physician’s] number one priority. But I’m fighting for my life, it’s MY number one priority, so I’d like the doctor I’m working with to feel almost the same.* – Man A.

With certain physicians, some men described feeling insecure. Sometimes, the man had sensed that the physician was trying to compel him to go with a specific option while aiming to make him believe that he himself was making the treatment decision. Perceiving the physician as knowledgeable and updated regarding treatments was important. Hence, the men assessed the competency of their physicians in various ways. Some asked candidly about the physician’s education and experience. Others described triangulating information between their online medical records, internet sources, other proficient persons, and the physician. The men also paid close attention to the physician´s wording and tone and described looking for cues that would help them interpret the communicated message.

*I came to the conclusion that they decided to start this … chemotherapy and whatnot on a 78-year old man, that must mean that they, so to speak, that they’re counting on it giving me a few more years with good quality of life.*

*I: Did you talk about that?*

*No, we didn’t, but that’s my own conclusion.* – Man C.

Several men emphasised that they preferred clear and direct communication about prognosis and treatment options to feel trust. However, they underlined that it was important to maintain hope while still getting honest answers.

### Navigating an unpredictable healthcare organisation

The men described how the healthcare organisation influenced their decision-making process. Some had experienced a smoothly operating system but when appointments or test results were delayed, they worried both about the result itself and about being forgotten altogether. Others thought their decision-making was unnecessarily delayed due to difficulties getting hold of the physician and feared that this was detrimental to their cancer treatment. When receiving new information, they wanted immediate action.

Most men had changed clinics, commonly from urology to oncology clinics, throughout their disease course. They wished for a cooperative approach between previous, current, and future healthcare providers to create a cohesive chain of care. Contrastingly, the organisation was experienced by others as unpredictable, slow and difficult to navigate, with the risk of information being overlooked when transferring between different departments or clinics. Some described feeling as if they had to pursue and coordinate their own care to hasten the path to a treatment decision. They tried to work around the system towards a treatment decision, by contacting potential physicians or clinics ahead of the referral or using personal connections for second medical opinions. One man concluded the healthcare as “too little, too late”, a theme that also emerged in similar ways in other men’s narratives:

*The only thing I kept hearing was that it was the holidays. That and ’we don’t have enough staff’. Well, I said ‘The cancer doesn’t take a holiday, it’s working full time around the clock, so that … you can’t say that to me’ […] I said to the doctor.* – Man B.

### Forming a basis for treatment decisions

#### Being guided by beliefs about cancer and cancer treatments

Several men voiced their beliefs about cancer and its treatments, guiding their decision-making in this disease stage. These stemmed from previous experiences (both their own and others’) and prior knowledge of cancer and cancer treatments. Prostate cancer was described as insidious, as it “learned” to avert treatments directed towards it, and opportunistic – a disease that seized an opportunity to grow when “the body was weakened” by other health issues. The men viewed cancer as illogical and inconsistent – its next move or response to treatment could never be predicted. Some claimed that they “knew nothing about cancer treatments” but simultaneously voiced beliefs about e.g., chemotherapy:

*…that was their concern with Docetaxel [chemotherapy], (…) it can’t be so tough that my natural resistance diminishes, (…) then the balance isn’t right, huh. And that isn’t easy, to attack the cancer but not attack me to the point of going under. You shouldn’t die from the chemo, if I’m gonna die from anything it should be the cancer.* – Man A.

This led to wishing to instead try less intrusive treatments, whereas others wanted to strike the cancer immediately with chemotherapy, that they believed to be the most effective treatment. Some expressed that they felt safe continuing with a treatment given that they knew it had been used before and been successful in others:

*You can say that, they have used chemotherapy before that … and for different illnesses and people have made it. (…) So why shouldn’t you believe in it today? I feel like you can almost dare to.* – Man D.

The men’s beliefs about cancer and its treatments seemed to be quite robust over the interviews, however, over time, a group of men started questioning their initial beliefs. The “truths” that once seemed obvious were gradually questioned by the men - for instance, one man described how his perception of cancer had started to change:

*Because cancer … if … it equals death, or it used to equal death once. (…) So if a person had gotten cancer it was … it was the end. But it’s not like that anymore.* – Man I.

#### Wanting and looking for personalised information

Quality and quantity of information were described as crucial to the men’s decision-making, and they also expressed preferences regarding types and timing of information. Some explained that they adamantly wished to know everything there was to know about current and future treatment options (e.g., expected benefits and possible side effects). Besides asking the physician, they accessed various sources such as internet websites (e.g., government healthcare service information and PC patient federations) to learn more and read about others’ experiences. Conversely, others described how too much information could be overwhelming and difficult to process, especially in stressing situations:

*She told me, this doctor, how it was and things. (…) And she said we would start a treatment and so on, and I didn’t really ask a whole lot at that point. Because you wanna take it piece by piece, you know? I couldn’t take that much in at the time.* – Man H.

For some, information preferences changed over time, as their questions arose:

*Now I want to … let some time pass. (…) You must try to ask the doctor questions and so, what is it one can expect from this, and those … those questions, they don’t come the first time you see a doctor, they come when you’ve got some background to ask.* – Man F.

The men expressed that when personal information needs had been met, this enabled them to partake in treatment decisions. For some, lack of appropriate information had been a consistent bother whereas others felt dissatisfied with the information from specific physicians or meetings. Common causes of frustration were conflicting information (between different physicians or other sources) or vague information that was difficult to interpret.

#### Waiting for a treatment evaluation

During or following treatment, the men described their waiting for treatment evaluation – a time characterized by uncertainty:

*Well, it’s a little … And what then … I haven’t yet come to … how this … chemotherapy works. If there’s a plan B if it [the cancer], so to speak, pops back up again, so that it starts to … be too active again? I’ll cross that bridge when I come to it.* – Man C.

Although uncertain, this phase was described as hopeful by some, whereas others experienced it as a worrying, stressful time preceding yet another new treatment decision or notification that no more treatments were possible. Some expressed faith in the medical advances in the PC treatment field and considered the availability of further treatments when evaluating and planning their treatments. They expressed a preference for treatments that would still keep other options open if their present treatment failed:

* You can put it like this, (…) now when you get the hormone treatment, maybe you feel a little calmer knowing that there is SOMETHING they can give, huh, that like … puts a stop to it. Nevertheless, (…) for everyone there’s a little … always a slight tworry, of what’s going to happen? (…) next time [next decision], what will it look like then?* – Man D.

## Discussion

The results from this study provide insights into men’s experiences of decision-making in life-prolonging treatments of mCRPC within the frame of an incurable illness. Over time, the men’s perspectives on the treatment outcome changed, and rather than wanting treatment at any cost some instead viewed their well-being as increasingly important. The results show that the men adapted and modified their decision-making roles and actions depending on the physicians they met and in relation to the organisation of healthcare. When striving to form a basis for treatment decisions, the men were sometimes guided by previous experiences and wanted information that was tailored to their needs regarding quality, quantity, and timing.

The men in this study had varying treatment decision-making role preferences and experiences, however, it was important for them that their role preferences were respected, regardless of what they preferred. Similarly, men’s decision-making role preferences have been shown to vary greatly also in men with localised PC [4, 5, 8, 9, 35]. Some men in this study expressed a lack of understanding of the value of their involvement in the decision-making process, which suggests a need for continuous discussions about their thoughts and preferences. Even if the different decision-making role preferences and experiences of the men in this study are in line with theoretical models (shared, paternalistic/physician-driven, informed/patient-driven) as described in the literature [36], the results also suggest that shared decision-making can manifest itself in different ways along a spectrum of levels of participation. Being informed about the treatment but ultimately leaving the decision to one’s physician was viewed as actively partaking in decision-making by the participants in this study, which in turn could be interpreted as a shared decision-making process. To achieve a shared decision-making process, it is maybe not necessary to urge a hesitant patient to make the decision themselves. Instead, staff must carefully explore the patient’s own view on what shared decision-making entails. This study also revealed that the men’s treatment decision-making role preferences are not all the same, nor are they fixed over time for each person. Instead, the men modify their decision-making approach and -role to the situation with the physicians they meet at the clinic appointments. For them, this meant sometimes assuming a different approach to, and role in, treatment decision-making than they had preferred. Some men had also experienced feeling compelled to assume a more driving role than they had wanted to, which has also been described in patients with localised PC [37]. Previous research has shown that a discrepancy between the preferred and actual decision-making role is associated with poorer health-related QoL, mood and physical health [38] which underlines the importance of exploring each man’s preferred role prior to each treatment decision. Further, men’s treatment decision-making role preferences are not regularly noted in their medical records, making it more difficult for the treating staff to comply with each man’s preferences. Routinely documenting how treatment decision-making has come about as well as the patient’s desired and actual role in treatment decision-making in the patient medical records might serve as a reminder and a way to keep the patient’s treatment decision-making role preferences on the agenda at each appointment at the oncology clinic. The men’s treatment decision-making role preferences also vary over time depending on the situation and physician, further underlining the need for their preferences to be explored not only once but continuously over time during their treatment course(s).

The results of this study show the significance of a trustful relationship with the treating physician, which was the most important relationship related to decision-making. This is much in line with previous research about decision-making in men with mPC [15]. However, even if the men in the present study should have had a designated contact nurse [39, 40] and treatment evaluation turned out to be part of the treatment decision-making process for men with mCRPC, nurses were not spontaneously mentioned by the men as parties in treatment decision-making. The reason for this is not known but it might indicate that nurses are an underutilised resource in this area that could provide the continuity that was asked for by the men in this study. The contact nurse’s tasks are e.g. to support patient participation in decision-making as well as to help with treatment evaluation [40]. The need for men with PC to have a designated nurse with specific knowledge and experience of PC has also been emphasised in previous research, such as a consensus article by Lamb et al. [22]. The article states that a nurse should be assigned to the patient as a contact point, and function as the patient advocate, aid in treatment decision-making in PC, and participate in follow-ups [22]. While a contact nurse could provide continuity in their healthcare for men with mCRPC, the contact nurse’s role in aiding in treatment decision-making needs to be further emphasized. Given that the contact nurse follows the man over time, while he awaits treatment evaluations and often continues to face new treatment decisions, the contact nurse could have an important function in e.g. the continuous use of decision aids. Decisional aids have been quite extensively studied and used among patients with localized PC [41–46] and less so in the later phases of the illness since the treatment panorama has evolved so rapidly. Each man’s individual wishes and priorities need to be put high on the agenda as they cannot be assumed, possibly with the help of decisional aids tailored for this late illness phase. These communication tools could be used as a starting point for discussions about the illness and its treatment options. Decisional aids could be used to establish both the patient’s treatment preferences and priorities but also his treatment decision-making role preferences as they may change. Given that they may vary over time, as seen in the present results, some sort of decisional aid could work as a way for clinicians to achieve a sense of how the men would like the treatment decision to be made to from appointment to appointment.

Our results showed that over time, the men’s perspectives on the treatment outcome changed, in the balancing act between prolonging life and their well-being and they expressed a need to talk about their priorities in life. The men’s wish for well-being has also been shown in previous research, highlighting the importance of incorporating aspects of QoL in decision-making in patients with advanced PC [15, 28] and other incurable cancers [26]. Still, our results also show that the patient’s perspective was individual and described as linked to how intrusive they had experienced previous treatments on their everyday lives. While several studies underline the importance of considering QoL specifically in treatment decision-making in cancer care [15, 26, 28], there is also research showing that patients across all stages of cancer may consider their QoL less important than the treating healthcare professionals do [27], further emphasizing the need for dialogue in order to understand each man’s unique priorities. These discussions should also comprise the man’s previous treatment experiences and its impact on everyday life, in order to capture what level of intrusion he might find acceptable in a future/upcoming treatment. In this late phase of PC, dialogue, communication and providing personalised information about life-prolonging treatments might be even more challenging and important than in earlier phases, as it involves numerous factors such as QoL, life expectancy, the patient’s wishes, and priorities that actually consider the remainder of the patients’ lives. This is alongside the increasing number of treatment options and sequencing possibilities. As the men’s wishes and needs change over time, even in this incurable phase, applying a more personalised approach from the whole care team in the care of men with mCRPC who are confronted with the gravity of their illness seems imperative.

### Methodological considerations

Several measures have been taken to achieve trustworthiness, as described by Graneheim et al. (2017) [33]. Through the purposive sampling strategy, we managed to achieve variation regarding the place of residency, age, relationship status, education level and type of first life-prolonging treatment. Even though variation in background characteristics does not guarantee variation in experiences, the sampling strategy was used as a way of strengthening credibility and transferability [33] by creating a sample with various background characteristics represented. The lack of variation regarding place of birth among the participants is however a limitation.

A strength is the number of interviews and the prospective design, which allowed us to follow some participants over the course of two years and up to five different life-prolonging treatment courses which yielded rich data and increased the understanding of their experiences regarding treatment decision-making. Only a few participants received a new treatment beyond their first one. Hence, the number of follow-up interviews are limited as each man’s treatment trajectory could not be foreseen at the time of inclusion. It is possible that follow-up interviews with the men who did not undergo additional treatments could have added further perspectives on decision-making. The sample size was continuously evaluated based on the interviews’ quality (depth and richness), utilizing the principle of information power [47]. Had the interviews been lacking in depth and richness, we would have had to increase the sample size in order to attain a sufficient amount of quality data. The interviews did, however, comprise rich and deep narratives, which enhanced information power in the data and thus, we decided not to recruit more participants. Another strength in the study is the open form of interview encouraging the men to narrate important dimensions of their experiences of treatment decision-making, while a limitation is that the participants were not explicitly asked to describe nursing input in relation to treatment decision-making. Data for the study was collected between 2016 and 2019, which may be a limitation given that the treatment landscape for mCRPC continues to evolve, However, there are different treatments represented in the sample, and since the study focuses on treatment decision-making regardless of specific treatment drugs, we believe that the results are still relevant and applicable in today’s mCRPC context.

To strengthen the dependability of the study results and avoid a single researcher’s preconceptions steering the analysis [33], all interviews were read by two authors and the analysis was performed collaboratively within the research group. The themes and subthemes were discussed in the entire research group on several occasions to achieve consensus. Further, methodological transparency, by use of quotations in the results, was used to strengthen credibility and create a basis for assessment of transferability and authenticity [33].

## Conclusions

In light of the results of the men’s experiences of decision-making in life-prolonging treatments of mCRPC, preferred treatment decision-making role and perspectives on the treatment outcome should be explored once mCRPC is diagnosed. These aspects should also be continuously addressed throughout their disease and treatment trajectory. Using a more personalised approach when caring for men with mCRPC could narrow the gap between what the men wish for and what they experience in terms of their role in treatment decision-making. This approach could also meet their individual needs and priorities. Future research needs to investigate how these patients’ decision-making can be further supported.

## Electronic supplementary material

Below is the link to the electronic supplementary material.

Supplementary Material 1

## Data Availability

The datasets generated and analysed during the current study are not publicly available due to the General Data Protection Regulations and the Swedish Ethical Review Act, but are available from the second author on reasonable request.

## Abbreviations

PC: Prostate cancer
mPC: Metastatic prostate cancer
mCRPC: Metastatic castrations-resistant prostate cancer
QoL: Quality of life
