# Supplementary material for: Men’s experiences of decision-making in life-prolonging treatments of metastatic castration-resistant prostate cancer – wishing for a process adapted to personal preferences: a prospective interview study
Source: BMC Med Inform Decis Mak. 2025 Mar 31;25:153. doi: 10.1186/s12911-025-02985-x (PMC11960004; doi:10.1186/s12911-025-02985-x)
Supplement: Supplementary file 1 — Supplementary Material 1 [file 12911_2025_2985_MOESM1_ESM.docx]

**Interview guide baseline interviews**

Opening question:

- Would you like to tell me about your situation with prostate cancer?

Themes to cover during the interview:

- Treatments received for prostate cancer?
- Participation in treatment decision-making?
- Treatment expectations?

**Interview guide follow-up interviews**

Opening question:

- Would you like to tell me how you have been since we last saw each other?

Themes to cover during the interview:

- Treatments received for prostate cancer?
- Participation in treatment decision-making?
- Treatment expectations?
